# Mid-term results of total hip arthroplasty with modified trochanteric osteotomy in Crowe type IV developmental dysplasia of the hip

**DOI:** 10.1186/s12893-020-01002-4

**Published:** 2021-01-06

**Authors:** Jing-Yao Jin, Taek-Rim Yoon, Kyung-Soon Park, Sheng-Yu Jin, Dong-Min Jung, Qing-Song Li

**Affiliations:** 1 Center for Joint Disease, Chonnam National University Hwasun Hospital, 160, Ilsim-Ri, Hwasun-Eup, Hwasun-Gun, Jeonnam Republic of Korea; 2 Department of Orthopedic Surgery, Center for Joint Disease, Yanbian Hospital: Yanbian University Hospital, Jilin Yanji, China

**Keywords:** Arthroplasty, Trochanteric osteotomy, Dysplasia, Development dysplasia of the hip, Crowe type IV

## Abstract

**Background:**

This study aimed to explore mid-term clinical results of cementless total hip arthroplasty (THA) with modified trochanteric osteotomy in Crowe type IV developmental dysplasia of the hip (DDH).

**Methods:**

Thirteen patients (13 hips) with Crowe type IV DDH who underwent THA with modified trochanteric osteotomy between May 2013 and October 2015 were retrospectively analyzed. The mean follow-up duration was 5.2 years (range, 4.9–6.1 years).

**Results:**

The mean Harris Hip Score (HHS) significantly (*p* < 0.05) improved from 30.7 (range, 22–38) to 87.5 (range, 83–93). The mean leg length discrepancy (LLD) was 53.4 mm (range, 42.1–68.5 mm) preoperatively. The final LLD was 5.6 mm (range, 2.4–9.1 mm; *p* < 0.05). The mean leg length after surgery was 47.4 mm (range, 33.6–67.2 mm) and the femur shortening distance was 43.8 mm (range, 31.2–53.4 mm). The average duration of bone union for the greater trochanter (GT) was 2.5 months (range, 1.5–3.6 months). There was no infection, GT non-union, or loosening (septic or aseptic) of the stem or cup in any case.

**Conclusions:**

THA with modified trochanteric osteotomy with a cementless cup is an effective treatment for Crowe type IV DDH. It can rebuild complex biomechanics and biology of hip dysplasia without increasing complications.

## Background

Developmental dysplasia of the hip (DDH) is the most common cause of secondary osteoarthritis of the hip. If patients are not treated adequately, they will have severe leg length discrepancy (LLD) and many other adulthood problems. In patients with DDH, the acetabulum is deficient anteriorly and superiorly. In cases of hypoplastic acetabulum, it is usually difficult to obtain enough bony coverage of the cup. In cases of high false acetabulum, it is generally recommended to place the cup in the true acetabular region to improve the long-term survival of the implant [1, 2].

Difficulties in reconstructing the femur include hypoplasia of the femur, excessive femoral anteversion, valgus neck-shaft angle, metaphyseal–diaphyseal mismatch, and posteriorly displaced greater trochanter (GT) [3]. Combining trochanteric osteotomy with proximal femur shortening can solve many of these problems. Many techniques, such as step-cut osteotomy, modified oblique femoral shortening osteotomy, double chevron osteotomy, transverse osteotomy, and shortening derotational subtrochanteric osteotomy, have been reported [4–8].

**Fig. 1:**
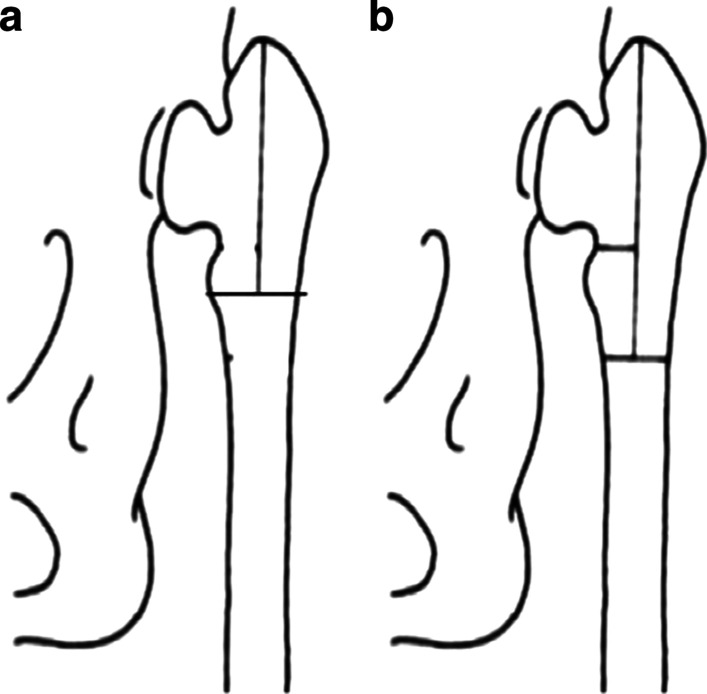
**a** Our osteotomy method. **b** Paavilainen et al.’s osteotomy method. Our osteotomy method was based on Paavilainen et al.’s osteotomy method with some modifications.

In 1990, Paavilainen et al. [9] performed femoral shortening osteotomy to treat 52 severely dysplastic and 48 totally dislocated patients with DDH. In this study, we have modified the method of osteotomy performed by Paavilainen et al. [9] (Fig. 1). In 2012, for the first time, Park et al. [10] treated 18 patients with severely dysplastic septic hip sequelae (SSH), but never used in patients with DDH Crowe type IV.

In this study, we used the same method to treat DDH Crowe type IV sequelae patients. We evaluated the outcome of LLD and osteotomy site union, and surgical technique with bulk bone autografts to define the effectiveness and safety of this procedure in DDH patients.

## Methods

Between April 2013 and October 2015, 13 patients (13 hips) underwent THA with modified trochanteric osteotomy. Of the 13 patients, three were men and 10 were women. All operations were performed by a single surgeon (Yoon TR). The average patient age at the time of surgery was 50 years (range, 31–68 years). Five patients received the procedure on the right side and eight patients had the procedure on the left side. The mean follow-up period was 5.2 years (range, 4.9–6.1 years); no patients were lost to follow-up (Table 1). All patients had Crowe group IV DDH according to the classification of Crowe et al. [11]. Preoperative radiography showed that all patients’ proximal femurs were straight and the femoral neck was lacking. This study was approved by the ethics committee of Chonnam National University Hwasun Hospital. Written informed consent was obtained from each patient. None of the patient had previously undergone any hip surgery before the procedure.

**Table 1:** Demographic details and preoperative status of patients

Age (years)	50 (range, 31–68)
BMI	22.6 (range, 19.14–25.92)
Follow up duration (years)	5.2 (range, 4.9–6.1)
Male	3
Female	10

*BMI* Body mass index

Medical history affecting the operation (such as heart disease and deep vein thrombosis), physical examination findings (such as Trendelenburg sign and limping gait), and radiographic findings (such as lumbosacral spine, lower limb, and pelvis) of all patients were assessed preoperatively. The acetabular diameter from anterior to posterior wall were estimated using 3D-computed tomography (CT). The location of the true acetabulum, level of femoral neck osteotomy, stem size, and cup size were estimated on anteroposterior and oblique hip radiographs using templated measurements.

All patients underwent surgery in a lateral decubitus position with a posterolateral approach. Provisional osteotomy was usually performed at the middle of the lesser trochanter. The femur was divided in medial and lateral two parts from apex of the GT, and the medial half of the part was removed. After adequate exposure, the true acetabulum was widened and deepened by the reamer. To avoid excessive reaming or acetabular wall fracture, reaming was performed mainly in superior and posterior directions where the bone stock was usually thick enough. After preparing the true acetabulum, the final metal cup (13 hips; Lima-L to, Udine, Italy) with ceramic on ceramic bearing (Biolox, Osteo AG, Selzach, Switzerland) was inserted. If the cup coverage was not satisfied, bulk bone autografts with resected femoral head were used to provide adequate coverage of the acetabular cup.

In all hips, Wagner cone prosthesis (13 hips; Zimmer, Winterthur, Switzerland) was used. If the proximal part of the femur was considered weak after femoral reaming, prophylactic cerclage wiring was used to avoid proximal fragment fracture during reaming of the medullary canal or insertion of the stem into the femur. The stem size was chosen during preoperative planning and intraoperatively considering soft tissue tension. If soft tissue was too tight with femoral stem trial, we did further reaming of the femoral cannal, seated the stem more deeply, or used one size smaller stem. And if reduction was still impossible because of contractures, the flexor and abductor muscles can be released from the iliac bone. Stems were inserted at 15° anteversion. After the final reduction, the GT was reattached to the proximal femur using ± cortical screws, cables, and a grip plate system (13 hips) (Dall Miles^®^, Stryker Orthopedics Inc., Mahwah, NJ, USA) with the hip abducted position.

Antibiotic prophylaxis was administered before surgery and at 1 or 2 days after surgery. Range-of-motion exercises were encouraged after 2–3 days of bed rest. Postopartively, the use of crutches was permitted, and complete weight-bearing was permitted only after obtaining radiographic confirmation of bone union. Active exercises were then strongly encouraged to stretch and strengthen the abductor muscles.

Preoperative data (such as those on hemoglobin levels), blood loss, transfusion requirement, surgical time, postoperative complication, and radiological and clinical outcomes were assessed in all patients. Total blood loss was defined as intraoperative blood loss and blood volume collected in the drain before removal. The drain was removed after 24 h with drainage of < 100 ml. The hemoglobin level was measured a day before surgery. The total number of transfusions, including packed red blood cells, fresh frozen plasma, and platelet concentrates used intraoperatively and postoperatively, was recorded. Surgical time was defined as the time from incision to complete wound closure. The details of preoperative paramiters are summarized in Table 2.

**Table 2:** Details of perioperative parameters

Operation time (min)	111.5 (range, 85–165)
Hemoglobin change after operation (mg/dl)	2.6 (range, 1.3–3.9)
Intraoperative blood loss (ml)	738.5 (range, 500–1000)
Total drainage (ml)	499.2 (range, 340–700)
Transfusion (No. of units)	3.3 (range, 2–5)

Postoperatively, all patients were followed up monthly until union was achieved at the osteotomy site and at 6 months, 1 year, and annually thereafter. At the postoperative visit, patients were evaluated using the Harris Hip Score (HHS). Radiological evaluations were performed for reviewing acetabular component anteversion, acetabular lateral opening angle, radiological leg length measurement, the stability of the femoral stem and acetabular component, and the time of the osteotomy site union.

Leg length discrepancy (LLD) was measured preoperatively and postoperatively using the method described by Sabharwal et al. [12]. To measure acetabular cup anteversion, we used the method described by Sah and Estok [13]. Stem subsidence was measured using the method described by Callaghan et al. [14]. To measure acetabular and stem location for the extent of radiolucency, we used the methods described by De Lee and Charnley [15] and Gruen et al. [16]. To evaluate stem and cup loosening, we used the methods described by Harris et al. [17] and Hodgkinson et al. [18]. Leg lengthening was measured using the method described by Park et al. [10].

All statistical analyses were performed using SPSS software package version 25.0 (SPSS Inc. Chicago, IL, USA). The two-sided, paired Student’s t-test was used to analyze preoperative and postoperative continuous variables. The data are presented as mean values with ranges. Statistical significance was set at p > 0.05.

## Results

The mean duration of surgery was 111.5 min (range, 85–165 min). Postoperative hemoglobin levels, intraoperative blood loss, total drainage, and transfusion requirements are shown in Table 2. For seven patients, the bulk bone autograft technique was used for adequate acetabular cup coverage. The average stem size was 15.2 (range, 13–17). In all cases, a grip plate was used for GT fixation. The results are summarized in Table 3.

**Table 3:** Details of components and fixation of greater trochanter

	No. of cases
Acetabular component	
Delta PF (Lima-Lto, udine, Italy)	13
Ceramic on ceramic bearing (Biolox, Osteo AG, Selzach, Switzerland)	13
Head size 28 mm	10
Head size 32 mm	3
Bulk bone autografts	7
Femoral component	
Wagner Conical stem (Zimmer, Winterthur, Switzerland)	13
Size 13	2
Size 14	3
Size 15	2
Size 16	3
Size 17	3
Average of stem size	15.2 (range, 13 to 17)
Greater trochanter fixation Grip plate (Dall-Miles)	13

At the last follow-up, the mean HHS was 87.5 (range, 83–93), which was significantly (*p* < 0.05) higher than the mean preoperative score of 30.7 (range, 22–38). The postoperative outcomes were excellent in 5 (38.0%) hips and good in 8 (62.0%) hips.

Preoperatively, all patients had a limp. However, no patient used a cane due to limping at the last follow-up. No cases showed an intraoperative fracture at the femur or acetabulum site. Intraoperatively, there were no gastrointestinal or cardiopulmonary complications. No patient developed postoperative nerve palsy.

The mean acetabular component anteversion angle was 17° (range, 16°–20°) and open angle was 37.1° (range, 29°–46°). Bony coverage over the socket was 83.8% (range, 85–100%). In seven patients, the bulk bone graft technique was used to increase cup coverage. Preoperatively, the mean LLD was 53.4 mm (range, 42.1–68.5 mm) and the mean leg lengthening was 47.4 mm (range, 33.6–67.2 mm). Postoperatively, there was a reduction to the mean LLD of 5.6 mm (range, 2.4–9.1 mm) (*p* < 0.05). The femoral shortening amount (43.8 mm [range, 31.2–53.4 mm]) was the overlapping length of greater trochanter reattached to the proximal femur.

**Fig. 2:**
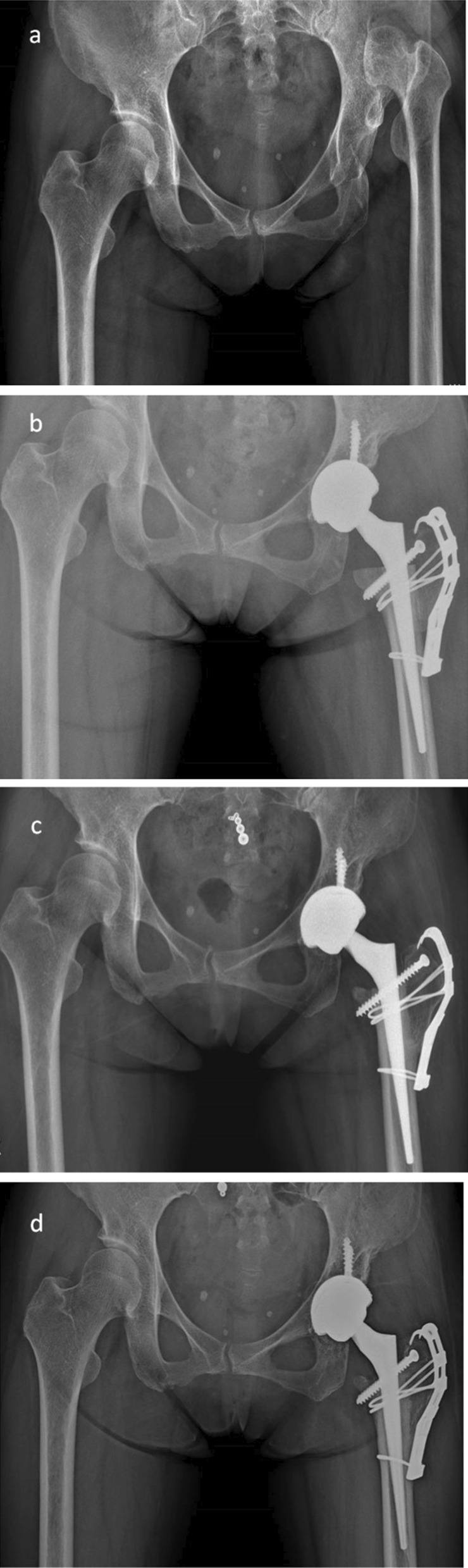
**a** Preoperative radiograph of a 36-year-old woman with Crowe type IV developmental dysplasia of the left-sided hip. **b** Bone union at the osteotomy site at 3 months postoperatively; no stem subsidence occurred. **c** Postoperative 1 year. **d** Postoperative 6 years

**Fig. 3:**
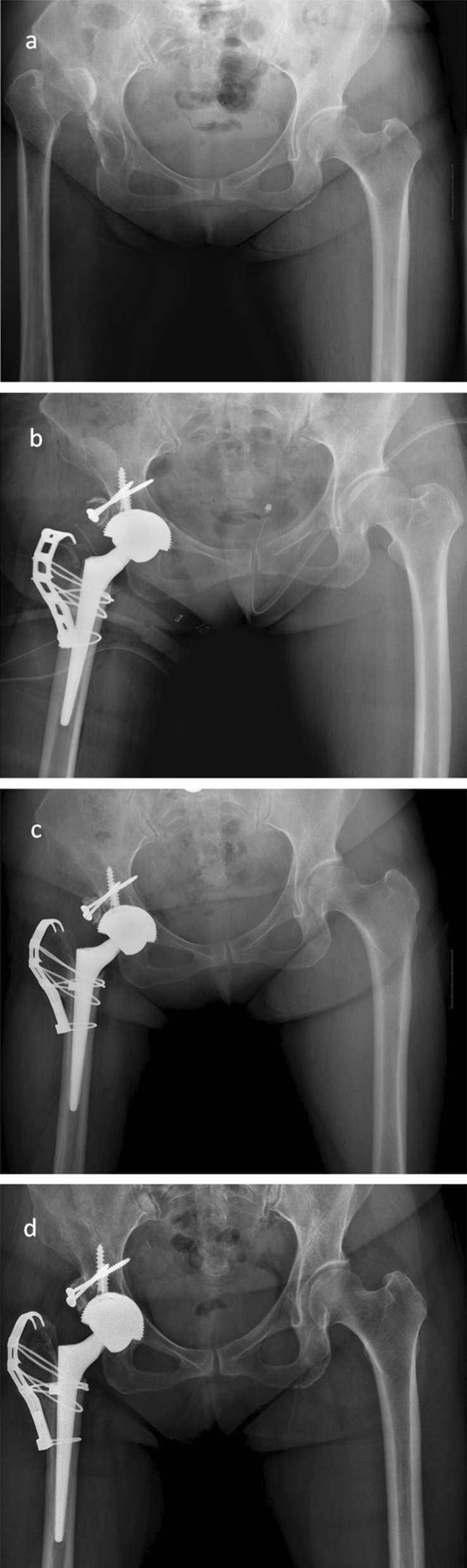
**a** Preoperative radiograph of a 36-year-old woman with Crowe type IV developmental dysplasia of the right-sided hip. **b** Bone union at the osteotomy site at 3 months postoperatively; no stem subsidence occurred. **c** Postoperative 1 year. **d** Postoperative 5 years

We reattached the proximal femur with (3 hips) (Fig. 2) or without (10 hips) (Fig. 3) cortical screws, cables, and grip plates in all patients. The mean time to GT union was 2.5 months (range, 1.5–3.6 months). There was no non-union at the osteotomy site in any patient. The details of clinical and radiologicl results are shown in Table 4.

**Table 4:** Details of clinical and radiological results

	Preoperative	Postoperative	p-value
HHS	30.7 (range, 22–38)	87.5 (range, 83–93)	< 0.05 < 0.05
LLD (mm)	53.4 (range, 42.1–68.5)	5.6 (range, 2.4–9.1)	
Leg lengthening (mm)		47.4 (range, 33.6–67.2)	
Leg shortening		43.8 (range, 31.2–53.4)	
Cup anteversion		17 (range, 16–20)	
Open angle		37.1 (range, 29–46)	
Duration of osteotomy site union (m)		2.5 (range, 1.5–3.6)	

*LLD* leg length discrepancy, *HHS* Harris Hip Score

*Statically significant *p* value < 0.05

No patient showed stem subsidence > 1 mm until the last follow-up. During follow-up, the radiolucent line between the stem and the femur was < 1 mm in all cases; one patient underwent stem revision due to a postoperative fall, which resulted in a periprosthetic fracture at the femur site. The region of osteolysis was limited in Zones 1 and 7. There was no aseptic or septic loosening in the acetabular and femur component. Postoperatively, no patient had a surgery-associated infection. One patient had a postoperative dislocation and was treated for closed reduction.

## Discussion

Many surgeons have described that it is challenging to perform THA in patients with DDH due to the deficient bone stock of the acetabulum. The most troublesome issue is acetabular reconstruction. Re-establishing the normal center of hip rotation is the most important thing in this field because non-anatomical placement of a component is an important predictor of acetabular loosening [19, 20]. It can have a direct impact on cup survivorship. In our study, all acetabular reconstructed in a true acetabulum and controlled fracture of the medial wall was performed to place the prosthetic acetabular component within the available iliac bone. No patients developed aseptic cup loosening postoperatively, and the survivorship of the cup was satisfactory.

A common complication in femoral shortening osteotomy is non-union at the osteotomy site. Previous studies have reported non-union rates ranging from 2.8 to 11.4% at the osteotomy site in hips with Crowe type IV DDH [4, 7, 21]. According to Hak et al. [22], the osteotomy site non-union rate is related to the rigidity of osteotomy site fixation. Step-cut, oblique, or chevron-shaped osteotomies [4–6] have inherent stability due to their three-dimensional geometry and increased bone healing surface. However, these osteotomies are technically complex and need surgical experience and prompt preoperative planning, especially for correcting femoral anteversion. In contrast, transverse osteotomy is easier to perform but it provides insufficient rotational stability at the osteotomy site [23]. Accoring to Wang et al. [24], the non-union rate is related to circumferential damage to the periosteum. Mainiating the osteoblastic activity of the periosteum can increase the bone union rate. In our study, the osteotomy site was in the middle of the lesser trochanter, and it was close to the proximal femur; however, in Paavilainen^’^s method, the osteotomy site was inferior to the lesser trochanter. Our osteotomy method may can save more host bone and preserve more soft tissue and periosteum attachment of proximal femur. After final reduction, we used grip plates and cables with ± cortical screws fixed at the osteotomy site. In our study, the duration of the osteotomy site union was 2.5 months (range, 1.5–3.6 months). No patient showed osteotomy site non-union. Our technique may provide better bone union at osteotomy sites. Further, it could lead to earlier weight bearing for patients.

The sciatic nerve injury occurs in 5.2% cases of THA for DDH [25]. There is no report on the maximum safe amount of leg lengthening for preventing significant sciatic nerve injury. Most previous studies have reported the range of leg lengthening as 3–4 cm [4–8, 21, 24, 26–28] and reported that a leg lengthening of > 4 cm indicated femoral shortening osteotomy to prevent nerve palsy. Rollo et al. [26] have combined two kinds of osteotomy in 15 patients (17 hips); the mean leg lengthening was 27 mm (range, 19–45 mm). There were two cases of nerve palsy. Wang et al. [29] performed transverse osteotomy to treat 62 patients (76 hips), and the mean leg lengthening was 39 mm (range, 30–55 mm). Postoperatively, there were three cases of nerve injury. In our study, if there is too much soft tissue tightness with femoral stem trial, we did further reaming of the femoral cannal, seated the stem more deeply, or used one size smaller stem. Hence, another osteotomy at the femur site for femoral shortening was not needed intraoperatively. Moreover, our osteotomy site was at the middle of the less trochanter; hence, more bone stock at the proximal femur was saved. In our study, the femur shortening was 43.8 mm (range, 31.2–53.4 mm) the leg lengthening was 47.7 mm (34.5–67.3 mm). No patient developed sciatic nerve palsy symptoms postoperatively (Table 5).

**Table 5:** Over view of relevant literature in the treatment of Crowe IV dysplasia combined with subtrochanteric femoral shortening osteotomy

	Patient (Hips)	DDH type	Osteotomy type	Follow up duration (y)	Pre LLD (mm)	Post LLD (mm)	Leg lengthening (mm)	Nerve injury	Osteotomy site union (m)	Non-union	Revision	Dislocation	Intraoperative fracture
Ozden et al. [4]	35 patients 45 hips	Crowe IV	Step-cut	10 (range, 7–14)	33 ± 25 (range, 0–75)	10 ± 6 (range, 0–25)	37 ± 14 (range, 21–47)	1	3.8 ± 0.7 (range 3–6)	3	4 stem 1 acetabular component	2 closed reduction 2 performed surgery	5
Kiliçoğlu et al. [5]	16 patients 20 hips	Crowe IV	Modified oblique femoral shortening	6.8 (range, 3.7–10.3)	45 ± 10 (range, 30–65)	10 (range, 0–30)	35 ± 17 (range, 0–58)	0	4 (range, 3–6)	1	1 stem 1 acetabular component	2 closed reduction 1 performed surgery	3
Li et al. [6]	18patients 22 hips	Crowe IV	Double chevron	6.5 (range, 5–10)	45 (range, 30–62	15 (range, 0–24)	38 (range, 25–60)	0	3 to 6 months	0	0	0	0
Zhu et al. [7]	20 patients 21 hips	Crowe IV	Transverse	3.3 (range, 2–5)	47 ± 11(range, 20 -70)	12 ± 4 (range, 0–20)	38 ± 9 (range, 30–55)	2	5 (range, 3–9)	1	1 stem	1 closed reduction	0
Charity et al. [8]	15 patients 18 hips	Crowe IV	Shortening derotational	9.5 (range, 4.3–14)	No details	No details	30 (range, 10–40)	1	No details	1	3 acetabular component	0	0
Yalcin et al. [21]	31 patients 44 hips	Crowe IV	Transverse	5.3 (range, 2–8	34 (range, 20–60)	22 (range, 10–32)	> 20, 6 patients < 20, 25 patients	27 (range, 19–45)0	4 (range, 2.5–14)	5	1 acetabular component	2 closed reduction	0
Wang et al. [24]	49 patients 56 hips	Crowe Type-III or IV	Transverse	10 (range, 4.8–14.3)	42 (range, 21–65)	11 (range, 6–14)	27 (range, 19–45)	3	6 (range, 4–9)	1	1 acetabular component	2 closed reduction 1 performed surgery	4
Rollo et al. [26]	15 patients 17 hips	Crowe IV	Transversal and Z-shaped	7.3 (range, 5.3–11.1)	45 (range, 38–90)	12 (range, 9–16)	39 (range, 30–50)	2	2.9 (range, 2.3–3.5)	0	0	0	1
Ollivier et al. [27]	24 patients 28 hips	Crowe IV	Transverse and step-cut	10 (range, 0.8–14.5)	43 (range, 26–60	6 (range, 0–20)	40 (range, 20–80)	0	10 (range, 2–28)	0	3 stem 1 acetabular component	2 closed reduction 1 performed surgery	5
Yasgur et al. [28]	8 patients 9 hips	Crowe IV	Transverse	3.6 (range, 2–7)	50 (range, 30–70	15 (range, 5–30)	35 (range, 30–55)	0	5 (range, 3–9)	1	1 stem	1 closed reduction	0
Wang et al. [29]	62 patients 76 hips	Crowe IV	Transverse	10 (range, 6.6–13.2)	43 (range, 21–65)	10 (range, 6–17)	22 (range, 12–38)	2	6 (range, 4–8)	1	1 stem 1 acetabular component	3 closed reduction	4
Baz et al. [32]	15 patients 21 hips	Crowe IV	Transverse	4.9 (range, 3–8)	58.19 (47–72)	No details	31.95 (21–45)	1 permanent	No details	No details	3 stem 1 acetabular component	2 performed surgery	5
Present series	13 patients 13 hips	Crowe IV	Modified trochanteric	5.2 (range, 4.9–6.1)	53.4 (range, 42.1–68.5)	5.6 (range, 2.4–9.1)	47.4 (range, 33.6–67.2)	0	2.5 (range, 1.5–3.6)	0	0	1 closed reduction	0

*DDH* developmental dysplasia of the hip, *LLD* leg length discrepancy

This study considered dislocation as an important complication, which may compromise the press fit between the femoral component and femoral fragment and result in disengagement and early loosening of the stem [7]. In previous studies, the dislocation rate was 0.92–2.93% [30, 31]. However, the postoperative dislocation rate in hips with Crowe type IV DDH was as high as 9.5–15.0% in some follow-up studies with the sample size of 9–28 hips [5, 27, 28, 32]. In our study, total dislocation rates were 7.6% that compared favorably with dislocation rates in these studies.

This study has some limitations. First, although our technique is reliable, a small number of patients were included in the study; hence, the study results should be interpretated with caution. Second, this study was based on two-dimensional plain radiographs. In some patients, the lower extremities were slightly rotated when the radiographs were taken, although could be measured accurately. Third, this study did not include a control group.

## Conclusion

In conclusion, our method may be easier to perform and provides earlier bone union at osteotomy sites and achieves similar leg lengths. Thus, THA with modified trochanteric osteotomy in Crowe type IV DHH is a safe and effective treatment procedure.

## Data Availability

The datasets used and/or analyzed in the current study are available from the corresponding author on reasonable request.

## Abbreviations

THA: Total hip arthroplasty
DDH: Developmental dysplasia of the hip
HHS: Harris Hip Score
LLD: Leg length discrepancy
